# Perceived self-efficacy on advance care planning in Latin America: ACP-SEs Latam validation

**DOI:** 10.3389/fpsyg.2026.1813541

**Published:** 2026-07-01

**Authors:** Patricia Bonilla, Tamara Rodríguez Quintana, Lester Wong Vázquez, Mohamed Sánchez, Serguei Iglesias-Moré, Stella Di Gennaro, Julia Fila, Verónica Veloso, Vilma A. Tripodoro

**Affiliations:** 1 Department of Health Sciences, Faculty of Health Sciences, Universidad Técnica Particular de Loja, Loja, Ecuador; 2 Department of Addiction Research, Fajardo Faculty, CEDRO Science, Technology, and Innovation Entity, University of Medical Sciences of Habana, Habana, Cuba; 3 PCA-Argentina Group, InPal Network, Pallium Institute Latin America, Buenos Aires, Argentina; 4 ATLANTES, Global Observatory of Palliative Care, University of Navarra, Pamplona, Navarra, Spain

**Keywords:** advance care planning, cross-cultural comparison, healthcare personnel, palliative care, self-efficacy

## Abstract

**Introduction:**

Advance Care Planning is a fundamental process in palliative and person-centered care, as it promotes clinical decisions that align with patients’ values and preferences. However, in Latin America no regionally validated instruments were available to measure professional self-efficacy in this domain although a locally validated Argentinian version is available.

**Objective:**

The objective of this study was to conduct a multicenter cross-cultural validation of the Perceived Self-Efficacy in Advance Care Planning Scale (ACP-SEsAR) among Latin American healthcare professionals, ensuring its cultural relevance and psychometric robustness.

**Methodology:**

An exploratory instrumental study was carried out with 685 healthcare professionals from 18 Spanish-speaking Latin American countries. Linguistic and cultural adaptation followed the International Test Commission guidelines. Twenty-one experts from 12 countries evaluated item clarity, coherence, sufficiency, and relevance using the Content Validity Coefficient (CVC). Reliability and construct validity were analyzed using Cronbach’s alpha, McDonald’s omega, and exploratory and confirmatory factor analyses.

**Results:**

The analyses demonstrated the scale’s strength and consistency in the Latin American context. The expert panel showed excellent agreement (CVC = 0.895). The scale exhibited high internal consistency (*α* = 0.974; *ω* = 0.93) and a solid unidimensional structure (KMO = 0.91; CFI = 0.96; RMSEA = 0.048).

**Conclusion:**

The Latin American version, termed ACP-SEs Latam, demonstrated excellent validity, reliability, and cultural adequacy, consolidating its role as an essential tool for strengthening professional training, evaluating interventions, and informing person-centered health policies.

## 1 Introduction

Shared Care Planning (SCP), also known internationally as Advance Care Planning (ACP), is a communicative and deliberative process through which individuals can express their values, preferences, and goals for future medical care, particularly when they may no longer be able to make decisions for themselves (Lasmarías et al., 2023) According to the international consensus supported by the European Association for Palliative Care (EAPC), ACP enables the definition and documentation of care goals, their discussion with family members and healthcare professionals, and their periodic review as the patient’s clinical condition evolves (van der Steen et al., 2025). This process fosters shared decision-making, improves communication between patients and healthcare teams, and promotes consistency between healthcare interventions and personal values (Lasmarías et al., 2023).

In ACP, the psychological component and emotional well-being constitute central dimensions, as anticipatory conversations about values and goals of care involve not only clinical decisions but also coping and emotional processing for patients, families, and healthcare professionals (Yue et al., 2026). In addition. ACP encompasses communicational, decisional, relational, ethical, and organizational dimensions, including shared decision-making, family involvement, documentation of preferences, and the alignment of care with patients’ values and goals (Killackey et al., 2020; Rosa et al., 2023; Yue et al., 2026). Evidence indicates that professional self-efficacy and family support are associated with greater willingness to engage in ACP and with decisions more closely aligned with personal values. This relationship is crucial because ACP relies on effective communication among patients, families, and healthcare professionals. Family support helps discussions about values, preferences, and future care. Professionals with greater self-efficacy are also more likely to start and sustain ACP conversations. This leads to better-aligned decisions with patients’ goals and preferences (Lasmarías et al., 2021a; Kishino et al., 2023; Tripodoro et al., 2024; Lyu et al., 2025). However, among healthcare professionals involved in palliative and end-of-life care, limited communication training and a low perceived competence in addressing end-of-life issues remain significant barriers to ACP implementation. These limitations may reduce professionals’ confidence in initiating conversations, exploring patients’ values and preferences, involving families, and supporting shared decision-making in complex clinical contexts.

In recent years, ACP has been recognized as an essential component of person-centered care and palliative care (Mas et al., 2021). In oncology, structured advance care planning has been associated with reduced aggressive end-of-life interventions, better patient–clinician communication and satisfaction, and care more consistent with patients’ preferred place and manner of death (Burghout et al., 2023; Levoy et al., 2023; Li et al., 2025). However, its systematic integration remains challenging, constrained by structural, cultural, and educational barriers, particularly in Latin American countries (Rocha Tardelli et al., 2023; Trevizan et al., 2026). The Latin American context continues to reveal significant inequalities (Tardelli et al., 2025).

A notable example of ACP implementation is the regional program developed in Catalonia, Spain. Through the standardization of guidelines, professional training initiatives, and quality indicators, this program has demonstrated how coordinated health system strategies can facilitate the integration of ACP into routine clinical practice (Lasmarías et al., 2023).

In Latin America, the integration of and access to palliative care remain limited and markedly unequal, despite estimates indicating that approximately 2.9 million people—including 110,000 children—require palliative care each year (Tripodoro et al., 2025). The level of palliative care development across the region provides a critical perspective on the capacity of national health systems to respond equitably to these needs. However, important differences exist between North America and Latin America regarding policy development, service provision, professional training, and access to essential medicines (Tripodoro et al., 2025).

In Latin America, the development and implementation of Advance Care Planning (ACP) remain heterogeneous. According to the Palliative Care Atlas of the Americas 2025, important disparities persist across countries in regulatory frameworks, professional training, service provision, and the integration of palliative care into health systems, limiting the systematic adoption of ACP in clinical practice (Tripodoro et al., 2025). While countries such as Chile and Uruguay have achieved more advanced levels of implementation supported by established legal frameworks, most countries are still in the early stages of development. Although recent progress has been reported in countries such as Ecuador and El Salvador, significant challenges remain regarding professional preparedness, awareness, and the availability of standardized procedures to support ACP implementation (Tripodoro et al., 2025).

Other relevant factors include prior ACP training, previous participation in ACP processes, positive perceptions of ACP benefits, professional role clarity, communication skills, family involvement, patient readiness, and access to structured tools that guide conversations and documentation (Lasmarías et al., 2021a; Yang et al., 2022; Murray et al., 2024; Zhou et al., 2024).

Evidence suggests that professionals with higher self-efficacy are more likely to initiate ACP conversations and support shared decision-making. Whereas lower self-efficacy may hinder engagement in these processes (Lasmarías et al., 2021a; Yang et al., 2022; Murray et al., 2024; Zhou et al., 2024). Consistent with these findings, recent qualitative and review studies have identified limited communication skills, lack of preparedness, emotional burden, and uncertainty or fear around end-of-life discussions as important barriers to ACP implementation (Poveda-Moral et al., 2021; Dias et al., 2023).

Given its influence on ACP implementation, measuring professional self-efficacy has become increasingly important. While previous efforts have assessed healthcare professionals’ confidence in ACP, the Advance Care Planning Self-Efficacy Scale (ACP-SE) developed by Baughman et al. (2017) is among the first rigorously validated instruments to evaluate professionals’ confidence across multiple domains of ACP practice.

Unlike broader measures that assess ACP knowledge, attitudes, or general preparedness, the ACP-SE specifically evaluates healthcare professionals’ confidence in conducting ACP conversations, exploring patients’ values and preferences, and supporting shared decision-making. This focus makes it particularly useful for assessing clinical readiness and evaluating the impact of educational and implementation initiatives (Baughman et al., 2017; Lasmarías et al., 2021a).

Despite these advances, Latin America still lacks standardized tools that adequately account for the region’s linguistic and cultural diversity. The heterogeneity of healthcare systems and the variability in palliative care training hinder cross-country comparisons and limit the development of common educational strategies (Pastrana et al., 2021). Without a regionally validated instrument, it is difficult to compare educational outcomes, evaluate implementation strategies, and generate evidence to support ACP policies across Latin America. In this context, the availability of an internationally and multicentrically validated scale represents a significant contribution to the region.

Because measurement instruments may not retain the same semantic, conceptual, and psychometric equivalence across different cultural and healthcare contexts, validation in one country cannot be assumed to apply automatically to other Latin American settings (Bartram et al., 2018; Coskun Benlidayi and Gupta, 2024; Alavi et al., 2026).

Although the ACP-SEs have been validated in Spain and subsequently adapted in Argentina, no multicountry psychometric evaluation has been conducted across Latin America. Given the region’s linguistic, cultural, educational, and healthcare system diversity, findings from a single-country validation cannot be assumed to apply uniformly across settings. Therefore, a multicenter validation is needed to establish the instrument’s reliability and applicability throughout Latin America (Bartram et al., 2018; Coskun Benlidayi and Gupta, 2024).

The primary objective of this study was to conduct a multicenter cross-cultural validation of the ACP-SEsAR Scale among healthcare professionals from Latin America. Additionally, its psychometric properties, including content validity, construct validity, reliability, and internal consistency, were evaluated to ensure its relevance and applicability across the region in clinical and educational settings.

## 2 Materials and methods

### 2.1 Study design and ethics

An exploratory instrumental study was conducted with healthcare professionals from different Latin American countries.

The study design followed the international guidelines of the International Test Commission for the adaptation and validation of psychometric instruments (Bartram et al., 2018) as well as the most recent recommendations on cross-cultural adaptation and structural validity of scales in the health sciences (Yang et al., 2022; Zhou et al., 2024; Alavi et al., 2026).

The study was approved by the Human Research Ethics Committee (CEISH) of the Universidad Técnica Particular de Loja (UTPL) under protocol No. 2024-02-INT-EI-RM-002, approved on March 20. 2024. Anonymity and data confidentiality were ensured, as well as voluntary and informed participation.

### 2.2 Participants and sampling

The study population consisted of 1.000 Latin American healthcare professionals registered in the educational events database of the Latin American Association for Palliative Care (ALCP) over the past 3 years. All participants had previously agreed to receive communications related to educational activities organized by the ALCP. Participation in this study was voluntary and required separate informed consent.

Sample size determination was based on psychometric recommendations for factor analysis rather than population proportion estimates. Considering that the ACP-SEs Latam scale comprises 19 items, a minimum ratio of 10 participants per item was established, requiring at least 190 participants, in accordance with contemporary recommendations for instrument validation studies (Memon et al., 2020). Additionally, larger samples yield more stable and interpretable factor solutions in exploratory and confirmatory factor analyses (Farag et al., 2025). The final sample of 685 healthcare professionals substantially exceeded these requirements, ensuring adequate statistical power for the psychometric evaluation of the instrument.

All eligible healthcare professionals registered in the ALCP educational activities database during the previous 3 years were invited to participate. Recruitment was based on voluntary response following electronic invitation and informed consent. A total of 685 professionals completed the survey and were included in the psychometric analyses, representing 68.5% of the eligible population.

**Figure 1:**
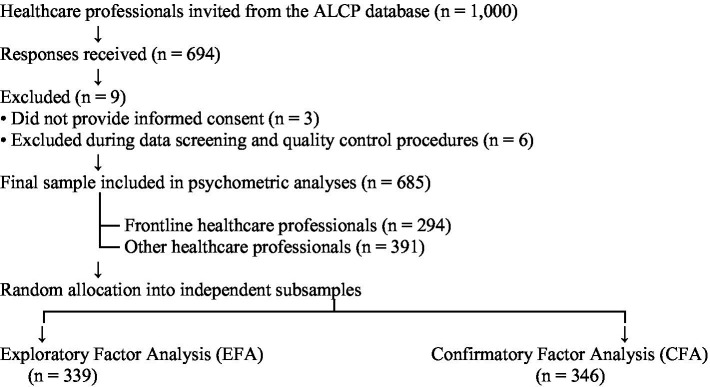
STROBE Flow diagram of participant recruitment and selection.

The participant recruitment and selection process is summarized in Figure 1. Of the 1,000 healthcare professionals invited to participate, 694 responses were received. Three respondents were excluded because informed consent was not provided, and six additional records were excluded during data screening and quality control procedures. The final analytical sample consisted of 685 healthcare professionals. Because the final analytical database did not retain a retrospective record of the specific exclusion categories applied during data cleaning, it was not possible to further distinguish exclusions due to incomplete questionnaires from those due to inconsistent or invalid responses.

Eligible participants were healthcare professionals (physicians, nurses, psychologists, social workers, physiotherapists, and related disciplines) practicing in Latin America who had previous training and/or professional experience in palliative care, advance care planning, serious illness communication, or end-of-life care. Participants were identified through the educational activities database of the Latin American Association for Palliative Care (ALCP) corresponding to the previous 3 years.

All eligible participants were allowed to participate in the study. However, questionnaires with missing responses, inconsistent answer patterns, or invalid data were excluded from the psychometric analyses.

### 2.3 Instrument

The ACP-SEsAR assesses healthcare professionals’ confidence in their ability to facilitate anticipatory conversations, explore values and preferences, and support shared decision-making in complex clinical contexts (Gennaro et al., 2024).

The instrument evaluated in this study was the ACP-SEsAR, a self-administered scale designed to assess healthcare professionals’ perceived self-efficacy in advance care planning. It consists of 19 items covering three key conceptual areas: (1) communication and exploration of patients’ values, preferences, and goals of care; (2) shared decision-making and discussions about future treatment preferences; and (3) documentation and implementation of agreed care plans. Responses are rated on a five-point Likert scale ranging from 1 (strongly disagree) to 5 (strongly agree). The instrument was derived from the original ACP-SEs developed by Baughman et al. (2017), subsequently translated and validated in Spain (Lasmarías et al., 2023) and culturally adapted in Argentina (Gennaro et al., 2024), where it demonstrated high reliability (Cronbach’s *α* = 0.89). The Argentine version served as the basis for the present multicenter Latin American validation, with María Eugenia Di Gennaro and Vilma Tripodoro, both co-authors of the Argentine adaptation and of the present study, contributing to the regional validation process.

### 2.4 Cultural and linguistic adaptation

The cultural and linguistic adaptation of the ACP-SEsAR Scale for Latin America was carried out in accordance with the International Test Commission Guidelines for Test Translation and Adaptation (Bartram et al., 2018) and the most recent methodological recommendations addressing the challenges of cross-cultural validation in clinical contexts (Alavi et al., 2026).

The process was structured in two phases: a qualitative expert-review phase and a psychometric statistical-validation phase. In the first phase, 21 experts from 12 Latin American countries participated. The panel size exceeded the minimum recommendations commonly proposed for content validation studies. Methodological literature suggests that expert panels ranging from 6 to 20 members are generally sufficient to achieve stable content-validity estimates, depending on the complexity of the construct and the heterogeneity of the target population (Schames Kreitchmann et al., 2024). The inclusion of 21 experts from 12 countries was intended to maximize cultural representativeness and strengthen the content validation process across Latin America. The sample was purposive and heterogeneous. Aiming to minimize group mean error and ensure professional diversity (Herrera Masó et al., 2022).

Consensus sessions were conducted virtually via videoconference (Zoom) in two iterative rounds. During each session, items were reviewed individually, and necessary modifications were discussed to achieve semantic equivalence, cultural relevance, and conceptual clarity. Opinions were collected anonymously and resolved through group consensus.

Each expert evaluated the items based on four criteria: sufficiency, clarity, coherence, and relevance, following the model proposed by Maldonado-Suárez and Santoyo-Telles (2024) and the methodological update by Spoto et al. (2025). A four-point ordinal scale was used (1 = does not meet the criterion; 4 = high level). Based on these evaluations, individual and overall Content Validity Coefficients (CVCs) were calculated according to the procedures proposed by Maldonado-Suárez and Santoyo-Telles (2024) and Schames Kreitchmann et al. (2024). This approach combined quantitative analyses of the experts’ ratings with qualitative interpretations of their observations, thereby providing a comprehensive assessment of content validity.

The consensus version resulting from the second round of expert review was considered culturally and linguistically adapted for the Latin American context and was subsequently subjected to psychometric evaluation. The final validated version of the ACP-SEs Latam was established following the exploratory and confirmatory factor analyses.

### 2.5 Statistical analysis and procedure

The statistical analysis aimed to evaluate the psychometric properties of the ACP-SEsAr Scale across three main dimensions: content validity, Reliability, and construct validity.

Content validity analysis was conducted through expert judgment, using the Content Validity Coefficient (CVC) to assess the level of agreement among judges regarding the clarity, coherence, relevance, and sufficiency of each item. Values equal to or above the commonly accepted threshold for this type of analysis were considered adequate; specifically, coefficients ≥ 0.80 were deemed acceptable. The results were complemented by a qualitative content analysis of the experts’ observations to identify patterns of convergence and divergence in item interpretation (Herrera Masó et al., 2022).

The internal reliability of the instrument was evaluated using Cronbach’s alpha (*α*) coefficients, with values ≥ 0.70 considered acceptable (Zakariya, 2022). Corrected item-total correlations were also calculated, along with the effect of deleting each item on the overall alpha value, to identify potential redundancies or internal weaknesses.

For construct validation, the total sample was randomly divided into two independent subsamples. The first subsample (*n* = 339) was used for the exploratory factor analysis (EFA), whereas the second subsample (*n* = 346) was used for the confirmatory factor analysis (CFA).

To examine construct validity, factor analyses were conducted in two stages. First, an exploratory factor analysis (EFA) was conducted using principal axis factoring to identify the scale’s latent structure and determine the optimal number of factors. Sampling adequacy was assessed using the Kaiser–Meyer–Olkin (KMO) measure, with values above 0.80 indicating meritorious adequacy, and Bartlett’s test of sphericity (*p* < 0.001; Hair et al., 2022). An oblique rotation (Promax) was applied because latent psychological constructs are often expected to correlate, allowing factors to associate freely if supported by the data. This approach facilitated the empirical evaluation of whether the instrument exhibited a unidimensional or multidimensional structure. Items with factor loadings below 0.40 were considered for removal.

Subsequently, a confirmatory factor analysis (CFA) was conducted using an independent subsample to verify the factorial structure identified in the EFA. Model fit was evaluated using the chi-square to degrees of freedom ratio (χ^2^/df), the Comparative Fit Index (CFI), Tucker–Lewis Index (TLI), Root Mean Square Error of Approximation (RMSEA), and Standardized Root Mean Square Residual (SRMR). Following commonly used guidelines for structural equation modeling, CFI and TLI values ≥ 0.90 and RMSEA and SRMR values ≤ 0.08 were considered indicative of acceptable model fit. These criteria were interpreted as heuristic guidelines rather than strict decision rules, taking into account the characteristics of the model and the study context (Hair et al., 2022; Kline, 2023).

Standardized factor loadings were analyzed to assess each item’s contribution to the construct. and Cronbach’s alpha of the final model was recalculated as an estimate of overall reliability, in accordance with the criteria proposed by Yang et al. (2022) and Zhou et al. (2024).

All analyses were performed using SPSS statistical software, version 24, and conventional psychometric criteria were applied to interpret validity and reliability indicators.

The final version of the scale was administered electronically via Google Forms after informed consent was obtained. Sociodemographic and professional variables were collected, including age, sex, country, profession, years of experience, and prior training in ACP. Participation was voluntary and confidential.

## 3 Results

### 3.1 Results of the cross-cultural adaptation and content validation

A total of 21 experts from 12 Latin American countries participated in the cross-cultural adaptation and content validation process. Their demographic and professional characteristics are summarized below, followed by the main modifications introduced to achieve semantic, linguistic, and cultural equivalence of the instrument.

The panel consisted of 15 women (71.4%) and 6 men (28.6%), with a mean age of 49.1 years and an average of 20.9 years of professional experience. Professions included physicians, nurses, psychologists, physiotherapists, social workers, and university faculty members from Bolivia, Colombia, Chile, Ecuador, El Salvador, Guatemala, Mexico, Panama, Paraguay, Peru, Uruguay, and Venezuela.

The review process resulted in modifications to 7 items to improve language neutrality, semantic clarity, and cultural appropriateness. Terms such as “determine” were replaced with “identify,” while “discuss” and “negotiate” were replaced with “reconcile.” Regional expressions were standardized, and locally specific acronyms were revised to enhance comprehension across Latin American contexts.

### 3.2 Participant characteristics

The final sample included healthcare professionals from diverse Latin American countries, disciplines, and levels of experience, providing a broad basis for the subsequent psychometric analyses.

A total of 685 healthcare professionals from 18 Latin American countries participated in the study: Argentina, Bolivia, Chile, Colombia, Costa Rica, Cuba, Ecuador, El Salvador, Guatemala, Honduras, Mexico, Nicaragua, Panama, Paraguay, Peru, the Dominican Republic, Uruguay, and Venezuela. The mean age of participants was 44.7 years (SD = 9.6), with a predominance of women (72%). The most represented professions were medicine (38%), nursing (32%), psychology (12%), social work (8%), physiotherapy (6%), and other health professions (4%). The average length of professional experience was 18.4 years (SD = 8.7), and 60% reported having received prior training in ACP. Table 1 presents the detailed sociodemographic characteristics, and Table 2 shows the distribution of participants by country.

**Table 1:** Sociodemographic characteristics of the sample (*N* = 685).

Characteristic	n (%) or Mean ± (SD)
Age (years)	44.7 **±** (9.6)
Sex	
Female	493 (72.0)
Male	192 (28.0)
Profession	
Physicians	260 (38.0)
Nursing	219 (32.0)
Psychology	82 (12.0)
Social work	55 (8.0)
Physiotherapy	41 (6.0)
Other health professions	28 (4.0)
Professional experience (years)	18.4 (8.7)
Prior ACP training	
Yes	411 (60.0)
No	274 (40.0)

**Table 2:** Distribution of participants by country (*n* = 685).

Countries	n	%
Ecuador	139	20.3
Argentina	112	16.4
Mexico	76	11.1
Chile	63	9.2
Colombia	47	6.9
Uruguay	47	6.9
Bolivia	40	5.8
Peru	34	5.0
El Salvador	30	4.4
Panama	27	3.9
Venezuela	20	2.9
Costa Rica	16	2.3
Guatemala	11	1.6
Nicaragua	6	0.9
Honduras	6	0.9
Dominicana	5	0.7
Paraguay	3	0.4
Cuba	3	0.4
Total	685	100.0

### 3.3 Content validity

Experts were selected according to the following criteria: (a) at least 5 years of professional experience in palliative care, advance care planning, bioethics, medical education, or related fields; (b) recognized academic or clinical expertise demonstrated through publications, teaching activities, leadership positions, or participation in scientific societies; (c) active professional practice in a Latin American country; and (d) willingness to participate in two rounds of expert review.

Qualitative feedback led to the revision of 7 items to improve clarity and linguistic neutrality, resulting in high agreement in item evaluations.

The content validity analysis yielded Content Validity Coefficient (CVC) values ranging from 0.83 to 0.96, with a global CVC of 0.895. indicating excellent inter-rater agreement. Most values were above the accepted threshold, indicating substantial consensus on the instrument’s sufficiency, clarity, coherence, and relevance. The overall CVC of the scale was rated as good, confirming that the items appropriately represent the theoretical construct of perceived self-efficacy in shared care planning. Detailed item-level and overall CVC results are presented in Table 3.

**Table 3:** Item-level and overall content validity coefficients (CVC) of the ACP-SEs Latam scale.

Items	CVCi
ACP1	0.911
ACP2	0.899
ACP3	0.911
ACP4	0.899
ACP5	0.905
ACP6	0.905
ACP7	0.869
ACP8	0.887
ACP9	0.911
ACP10	0.905
ACP11	0.902
ACP12	0.869
ACP13	0.884
ACP14	0.887
ACP15	0.881
ACP16	0.905
ACP17	0.896
ACP18	0.887
ACP19	0.890
Overall CVC	0.895

CVCi, Item-Level Content Validity Coefficient. CVC values ≥0.80 were considered acceptable; values ≥0.90 indicate excellent content validity.

Qualitative analysis of the experts’ observations led to terminological adjustments in 7 items, primarily aimed at improving semantic and cultural equivalence. Technical or hierarchically connoted terms were replaced with more neutral and accessible expressions suitable for Latin American clinical contexts, thereby ensuring universal comprehension of the content.

### 3.4 Construct validity

The Exploratory Factor Analysis (EFA) revealed a clear unidimensional structure. The Kaiser–Meyer–Olkin (KMO) index was 0.91, indicating excellent sampling adequacy, and Bartlett’s test of sphericity was significant (χ^2^ = 2587.4; *p* < 0.001), confirming the suitability of the correlation matrix for factor analysis. Results of the exploratory factor analysis, including eigenvalues and explained variance, are presented in Table 4.

**Table 4:** Results of the exploratory factor analysis: eigenvalues and explained variance.

Factor	Initial eigenvalues			Extraction sums of squared loadings		
	Total	% of variance	Cumulative %	Total	% of variance	Cumulative %
1	12.859	67.681	67.681	12.535	65.975	65.975
2	0.887	4.670	72.351			
3	0.681	3.586	75.937			
4	0.636	3.346	79.283			
5	0.513	2.702	81.985			
6	0.442	2.329	84.314			
7	0.382	2.010	86.324			
8	0.354	1.863	88.187			
9	0.318	1.672	89.860			
10	0.301	1.584	91.444			
11	0.272	1.433	92.877			
12	0.224	1.177	94.054			
13	0.203	1.066	95.120			
14	0.195	1.025	96.145			
15	0.184	0.966	97.111			
16	0.160	0.842	97.954			
17	0.147	0.776	98.729			
18	0.123	0.649	99.378			
19	0.118	0.622	100.000			

Extraction method: principal axis factoring.

The first factor showed an eigenvalue of 12.86 and explained 67.68% of the total variance. No additional factors exceeded the Kaiser criterion (eigenvalues> 1), further supporting the instrument’s unidimensionality. Factor loadings ranged from 0.608 to 0.888, indicating substantial contributions of all items to the underlying construct (Table 5).

**Table 5:** Descriptive item statistics and factor loadings.

Ítems	Mean	SD	Skewness	Kurtosis	Factor loading
ACP1	3.79	1.053	−0.530	−0.490	0.795
ACP2	3.81	1.014	−0.546	−0.424	0.843
ACP3	3.71	1.026	−0.381	−0.643	0.877
ACP4	3.82	1.003	−0.571	−0.345	0.819
ACP5	3.85	1.015	−0.611	−0.416	0.835
ACP6	3.77	1.137	−0.640	−0.417	0.758
ACP7	3.70	1.026	−0.423	−0.610	0.858
ACP8	3.71	1.045	−0.501	−0.560	0.851
ACP9	4.12	0.984	−0.913	0.014	0.712
ACP10	3.69	1.157	−0.547	−0.547	0.608
ACP11	3.38	1.255	−0.240	0.132	0.725
ACP12	3.55	1.099	−0.367	−0.610	0.853
ACP13	3.70	1.033	−0.576	−0.201	0.876
ACP14	3.99	0.995	−0.785	−0.097	0.888
ACP15	3.87	1.038	−0.663	−0.259	0.854
ACP16	4.21	0.900	−1.081	0.688	0.795
ACP17	3.92	1.020	−0.725	−0.150	0.767
ACP18	3.83	1.002	−0.651	−0.163	0.854
ACP19	3.72	1.042	−0.455	−0.460	0.810

Exploratory factor analysis was conducted using principal axis factoring.

The Confirmatory Factor Analysis (CFA) conducted on an independent subsample corroborated the single-factor structure, with standardized factor loadings above 0.50 for all items and satisfactory overall model fit, as indicated by the χ^2^/df, CFI, TLI, SRMR, and RMSEA indices. Results of the confirmatory factor analysis and model fit indices are presented in Table 6.

**Table 6:** Goodness-of-fit indices of the unidimensional model.

Index	Obtained value	Reference criterion
χ^2^/df	1.87	≤ 3.0
CFI	0.96	≥ 0.90
TLI	0.95	≥ 0.90
RMSEA	0.048	≤ 0.06
SRMR	0.041	≤ 0.08

Reference criteria based on Kline (2023).

The goodness-of-fit indices indicated excellent model performance, with CFI and TLI values above 0.95 and RMSEA and SRMR values below recommended thresholds, providing robust support for the adequacy of the unifactorial model. The scale, therefore, demonstrated strong construct validity, supporting the theoretical hypothesis that perceived self-efficacy in advance care planning is a unidimensional construct.

### 3.5 Internal reliability

The reliability analysis demonstrated high internal consistency, with a Cronbach’s alpha (*α*) of 0.974 and a McDonald’s omega (*ω*) of 0.93. Corrected item–total correlations ranged from 0.64 to 0.88. Detailed reliability statistics for each item are shown in Table 7.

**Table 7:** Item–total correlations and Cronbach’s alpha if item deleted.

Item	Corrected item-total correlation	Cronbach’s alpha if item deleted
ACP1	0.780	0.972
ACP2	0.827	0.972
ACP3	0.853	0.972
ACP4	0.795	0.972
ACP5	0.834	0.972
ACP6	0.743	0.973
ACP7	0.847	0.972
ACP8	0.841	0.972
ACP9	0.737	0.973
ACP10	0.642	0.974
ACP11	0.730	0.973
ACP12	0.836	0.972
ACP13	0.875	0.971
ACP14	0.872	0.971
ACP15	0.860	0.971
ACP16	0.805	0.972
ACP17	0.758	0.973
ACP18	0.849	0.972
ACP19	0.807	0.972
Total	0.974	

Overall Cronbach’s alpha = 0.974.

The scale demonstrated excellent internal consistency, with a global Cronbach’s alpha coefficient exceeding the values considered optimal for psychological measurement instruments. All items contributed positively to the instrument’s homogeneity, and corrected item–total correlations remained within the expected range. Corrected item–total correlations ranged from 0.64 to 0.88, indicating adequate discrimination and consistency across items. No item showed a significant increase in alpha when deleted, indicating strong internal coherence across the 19-item set. Consequently, all 19 items were retained in the final version of the ACP-SEs Latam scale.

These results support the instrument’s reliability and suggest that the ACP-SEsAR consistently measures perceived self-efficacy in shared care planning across diverse healthcare professional profiles.

## 4 Discussion

This study aimed to conduct a cross-cultural adaptation and psychometric validation of the ACP-SEs Latam Scale among healthcare professionals in Latin America, evaluating content validity, internal reliability, and construct validity within a multicenter sample spanning 18 countries. In summary, we found high inter-rater agreement, excellent internal consistency, and a unidimensional structure with good fit in both EFA and CFA. These findings address the central research question, whether the ACP-SEs Latam is valid and reliable in the Latin American context, and help close an important knowledge gap.

A major strength of this study is its multicenter design, which included healthcare professionals from 18 Latin American countries and expert reviewers from multiple national contexts. This broad participation enhances the regional representativeness of the findings and supports the applicability of the ACP-SEs Latam across diverse healthcare systems and cultural settings. To the best of our knowledge, no previous Spanish-language version had undergone a multicenter regional validation integrating countries with diverse income levels and healthcare cultures.

Our results are consistent with the original development of the ACP-SEs (Baughman et al., 2017). supporting the conceptual invariance of the “ACP self-efficacy” construct across clinical and cultural contexts. In the original scale, the exploratory factor analysis already suggested a single-factor structure with excellent internal consistency, similar to that observed in the present study, thereby reinforcing the hypothesis of a core construct integrating conversational skills, the management of values and preferences. and the clinical documentation of ACP. The very high internal consistency observed in the present study further supports the coherence of the construct across Latin American contexts. Although Cronbach’s alpha coefficients above 0.95 may sometimes suggest potential item redundancy, the corrected item-total correlations and factor loadings indicated that all items contributed meaningfully to the construct. Therefore, despite the high internal consistency observed, there was no empirical evidence supporting item elimination.

Although the items were originally designed to cover three conceptual domains of ACP practice, the factor analyses suggest that these domains operate as manifestations of a broader latent construct of perceived ACP self-efficacy, supporting a parsimonious unidimensional interpretation. The overall Content Validity Coefficient (CVC = 0.895) indicates a high degree of expert agreement regarding the relevance, clarity, coherence, and sufficiency of the items. This finding supports the adequacy of the cross-cultural adaptation process and suggests that the content of the ACP-SEs Latam is conceptually appropriate across diverse Latin American healthcare contexts.

Likewise, our findings are consistent with recent validations and developments. The Argentine validation of the ACP-SEs demonstrated high Cronbach’s alpha and utility for discriminating among professional groups (Gennaro et al., 2024). In parallel, the Chinese adaptation of a three-dimensional scale reported adequate validity and reliability (Yang et al., 2022), underscoring that, beyond structural variations across contexts. ACP self-efficacy is both measurable and clinically relevant. Regarding related instruments, the development of an ACP practice preference scale in nursing confirmed a consistent structure and good reliability (Yang et al., 2023). Similarly, a new ACP self-efficacy instrument based on the Transtheoretical Model achieved high internal consistency and the expected associations with stages of change (Murray et al., 2024).

Beyond psychometric metrics, the clinical relevance of self-efficacy has been documented both in scale development studies involving family physicians (Baughman et al., 2017) and in intervention and qualitative research (Oji et al., 2022). However, not all interventions produce additional effects on self-efficacy: a cluster-randomized controlled trial in primary care found no significant differences compared with usual care (Stevens et al., 2024), suggesting that prior awareness and contextual factors may modulate outcomes. In this regard. The availability of a valid regional measure. Such as the ACP-SEs Latam, enables stratifying training needs and evaluating intermediate outcomes in educational programs. In another study conducted in Argentina using the ACP-SEsAR scale, half of the professionals who participated in ACP processes showed a significant increase in their scores, up to 7.5 points higher than those who did not. Differences between physicians and non-physicians highlighted areas for improvement related to communication skills, role and task definition, and legal aspects (Tripodoro et al., 2024).

Our contribution is also methodological. The adaptation followed contemporary standards for cross-cultural adaptation in multinational studies (Coskun Benlidayi and Gupta, 2024), emphasizing semantic and conceptual equivalence and expert judgment through the Content Validity Coefficient (CVC). The high acceptability and psychometric performance observed support the usefulness of the ACP-SEs Latam for diagnosing training needs, monitoring the impact of educational programs, and serving as a process indicator in ACP implementation strategies. The link between self-efficacy and clinical practice, observed among primary care professionals in Spain (Lasmarías et al., 2021b), further reinforces these applications. Although the instrument showed stable psychometric performance in a multicountry sample, future studies should formally evaluate measurement invariance across countries and professional groups to confirm the equivalence of score interpretation throughout the region.

The findings suggest prioritizing educational interventions focused on conversational skills, the management of complex situations, and documentation. Using the ACP-SEs Latam as a change-sensitive outcome measure.

The ACP-SEs Latam demonstrates solid validity and reliability and emerges as a strategic, culturally adapted tool for research, training, clinical evaluation, and quality improvement in ACP across Latin America. By providing a standardized measure of professional self-efficacy, this instrument helps close an important regional gap in the assessment of ACP-related competencies.

In palliative care, advance care planning self-efficacy is particularly important because many healthcare professionals, despite recognizing the need to address these issues, do not initiate ACP conversations due to a lack of confidence in their communication and clinical skills. Higher levels of self-efficacy have been associated with a greater frequency of ACP discussions, more consistent documentation of patients’ preferences, and better alignment of care with patients’ values and goals (Baughman et al., 2017; Gilissen et al., 2020; Graupner et al., 2021; Lasmarías et al., 2021b).

Low self-efficacy may lead healthcare professionals to avoid or postpone conversations about prognosis, personal values, and end-of-life decision-making, thereby perpetuating gaps between patients’ preferences and the care they ultimately receive (Baughman et al., 2017; Gilissen et al., 2020; Lasmarías et al., 2021b). Studies involving physicians and nurses have shown that self-efficacy, even more than theoretical knowledge alone, is associated with greater engagement in advance care planning practices, including initiating conversations, completing advance directives, and documenting patients’ care preferences (Gilissen et al., 2020; Graupner et al., 2021; Lasmarías et al., 2021b).

The Advance Care Planning Self-Efficacy Scale, adapted and validated in Latin America, provides a reliable, unidimensional measure of healthcare professionals’ confidence in performing advance care planning activities. It allows for the identification of professional groups and clinical settings with lower levels of self-efficacy, facilitates the analysis of associations between self-efficacy and factors such as prior training in advance care planning, experience, and participation in specialized teams, and enables the evaluation of the impact of advance care planning training and implementation programs on the confidence and competence perceived by professionals in clinical practice (Baughman et al., 2017; Lasmarías et al., 2021b; Yang et al., 2022; Zhou et al., 2024; Chen et al., 2025).

### 4.1 Strengths and limitations

This study is strengthened by its Latin American multicenter scope, rigorous content validation, and confirmation of a unidimensional structure. However, the use of convenience sampling, self-reported measures, and a cross-sectional design may limit generalizability and preclude assessment of temporal stability and causality. Furthermore, metric invariance across countries and professional subgroups and test–retest reliability were not examined. It is important to note that the psychometric evaluation of the scale is not yet exhaustive, as convergent, discriminant, and criterion validity analyses were not included at this stage. Moreover, because recruitment relied on a highly specialized sample linked to palliative care networks, there may be restricted score variability and a potential ‘ceiling effect’, which limits the applicability of these findings to professionals less familiar with advance care planning. Finally, stratified analyses by country, profession, or level of experience were not conducted in this study. Future studies should address these gaps through longitudinal designs, multigroup analyses, and educational intervention studies to better understand regional applicability and differences across subgroups. In addition, the uneven distribution of participants across countries may have influenced the representativeness of some national contexts.

## 5 Conclusion

The multicenter cross-cultural validation of the ACP-SEs Latam Scale represents a meaningful contribution to Latin America by providing, for the first time, a standardized and culturally appropriate measure of professional self-efficacy in shared care planning. The scale demonstrated excellent reliability, adequate content validity, and confirmed unidimensional construct validity. Its strong internal consistency, clear factorial structure, and conceptual correspondence with previous versions developed in Spain and Argentina support its reliable use across diverse clinical and educational settings in the region.

Beyond its methodological value, this study delivers a practical tool to enhance healthcare workforce training, evaluate the impact of educational interventions, and monitor the quality of value-based clinical conversations with patients. Collectively, the ACP-SEs Latam emerges as a strategic resource for advancing more competent, humane, and person-centered palliative care throughout Latin America.

In addition, the findings open avenues for future research to examine the instrument’s cultural and professional invariance, as well as its sensitivity to change following advance care planning training. From a health policy perspective, the availability of a validated, standardized scale enables the generation of comparable indicators of care quality, which are valuable for developing national guidelines and implementing palliative care programs.

## Data Availability

The datasets presented in this study can be found in online repositories. The names of the repository/repositories and accession number(s) can be found at Mendeley Data, doi: 10.17632/x974p7g8w4.1.
